# Long Noncoding RNA *LncPGCR* Mediated by TCF7L2 Regulates Primordial Germ Cell Formation in Chickens

**DOI:** 10.3390/ani11020292

**Published:** 2021-01-24

**Authors:** Jingyi Jiang, Chen Chen, Shaoze Cheng, Xia Yuan, Jing Jin, Chen Zhang, Xiaolin Sun, Jiuzhou Song, Qisheng Zuo, Yani Zhang, Guohong Chen, Bichun Li

**Affiliations:** 1 Key Laboratory of Animal Breeding Reproduction and Molecular Design for Jiangsu Province, College of Animal Science and Technology, Yangzhou University, Yangzhou 225009, Jiangsu, China; jiangjy1127@163.com (J.J.); carolchen1996@163.com (C.C.); chengsz258@163.com (S.C.); yuanxia0306@gmail.com (X.Y.); mx120170642@yzu.edu.cn (J.J.); m160647@yzu.edu.cn (C.Z.); sunxiaolin19@sina.cn (X.S.); 006664@yzu.edu.cn (Q.Z.); ynzhang@yzu.edu.cn (Y.Z.); ghchen@yzu.edu.cn (G.C.); 2 Animal & Avian Sciences, University of Maryland, College Park, MD 20741, USA; songj88@umd.edu

**Keywords:** chicken (*Gallus gallus*), LncRNA, primordial germ cell, TCF7L2, *Btrc*, ceRNA

## Abstract

**Simple Summary:**

The potential of primordial germ cells (PGCs) for multidirectional differentiation, together with their unique regeneration ability, makes them one of the most promising seed cells in clinical medicine and tissue engineering research. However, not enough PGCs can be obtained to meet the demand, which limits their application. We defined a novel long noncoding RNA (lncRNA) mediated by epigenetics, which could activate the miR-6577-5p/*Btrc* pathway to promote the formation of PGCs. The technical system we have established is a useful tool to obtain sufficient PGCs for scientific research. Our study offers great theoretical and practical value in the production of transgenic animals or genomic imprinting in poultry. We believe that our study will help researchers in the fields of agricultural production, developmental biology, and cell biology.

**Abstract:**

Although lncRNAs have been identified as playing critical roles in the development of germ cells, their potential involvement in the development of PGCs in chickens remains poorly understood. Differentially expressed lncRNAs (DELs) from previous RNA-seq of embryonic stem cells (ESCs), PGCs, and spermatogonial stem cells (SSCs) were analyzed by K-means clustering, from which a key candidate, lncRNA (lncRNA PGC regulator, *LncPGCR*) was obtained. We confirmed that *LncPGCR* plays a positive role in the development of PGCs by increasing the expression of the PGC marker gene (*Cvh* and *C-kit*), while downregulating the pluripotency-associated gene (*Nanog*) in vitro and in vivo. The activation and expression of *LncPGCR* are regulated by histone acetylation, and transcription factor TCF7L2. Mechanistically, a rescue assay was performed to further confirm that *LncPGCR* contributed to the development of PGCs by regulating the gga-miR-6577-5p/*Btrc* signaling pathway. Adsorption of gga-miR-6577-5p activated the WNT signaling cascade by relieving the gga-miR-6577-5p-dependent inhibition of *Btrc* expression. Taken together, our study discovered the growth-expedited role of *LncPGCR* in PGCs development, showing the potential *LncPGCR*/miR-6577-5p/*Btrc* pathway. The results and findings provide a novel insight into the development of PGCs.

## 1. Introduction

Chicken primordial germ cells (PGCs) would be invaluable for in vitro studies of aspects of chicken embryogenesis. The multidirectional differentiation potential and unique migration pathway are chicken PGCs’ major biological properties, which can be applied to generate genetically modified poultry and conserve poultry’s genetic resources [1]. The induction of paracrine signaling [2,3], inhibition of somatic fate [4], alteration of epigenetic marks [5], and maintenance of pluripotency [6] are important to the development of PGCs. Considerable effort has been devoted to maintaining and propagating chicken PGCs in vitro. However, the limited number of PGCs which can be obtained greatly limits their clinical application. Therefore, in-depth exploration is needed of the development mechanism of PGC, to overcome the limitations, and improve our technical ability to effectively cultivate or induce PGCs in vitro.

**Figure 1:**
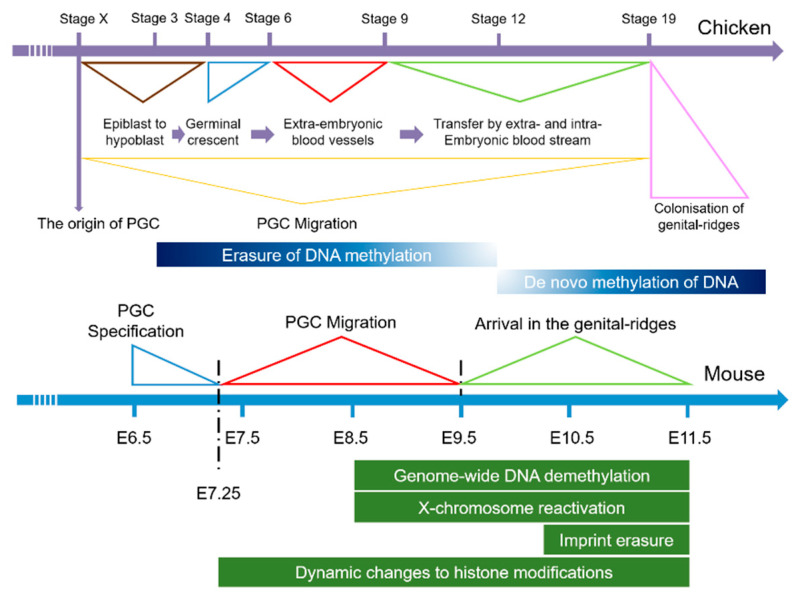
Development of mouse and chicken primordial germ cells.

Since the discovery of PGCs, researchers have devoted extensive attention to the study of their developmental mechanism, which is a complex and sophisticated regulatory process, affected by many factors (Figure 1). Studies have shown that genes [4,7,8,9], signal pathways [10,11], and growth factors [12,13] all play important roles in the development of PGCs in mice, and research into their mechanism has gradually turned to the field of epigenetics [14]. The development of PGCs has been subjected to an epigenetic reprogramming process that is genome-wide [15,16], including genome-wide DNA methylation erasure, chromosome inactivation, histone modification, and transposon silencing [17]. Current research on chickens shows only that genes such as *Wnt5A*, and *FGF8* may regulate the formation of their primordial germ cells [18,19,20]. However, the efficiency of inducing the production of PGCs in vitro has not yet achieved the expected levels. Therefore, there is an urgent need for innovative exploration of the developmental mechanism of chicken PGCs from new directions.

The recent release of an ultra-high-throughput approach to the sequencing of RNA (RNA-seq) has paved the way for identification and annotation of numerous long noncoding RNAs in the chicken genome [21]. Also, a quantity of binding sites for PGC marker genes (*Blimp1/Prdm1*) were found to be related to lncRNA in mouse PGCs [7]. Therefore, lncRNAs have recently come to the forefront as functional molecules involved in cell differentiation by interacting with microRNA [22], recruiting proteins as scaffolds to form complexes [23], and trapping transcription factors [24,25]. While different expression positions of LncRNA exhibit distinct molecular mechanisms [26,27], the specific regulatory mechanism of LncRNA during the differentiation of ESCs into PGCs remains unclear.

Here, we provide evidence that LncRNAs intimately regulate PGC development through a novel network of functional interaction between lncRNAs and miRNAs. In this study, we have identified a novel PGC-specific lncRNA (*LncPGCR*, TCONS_00948124) in RNA-seq analysis results of genes up-regulated in PGCs. We found that increased LncPGCR levels were activated by the transcription factor TCF7L2, and, histone acetylation. Moreover, *LncPGCR* was also shown to regulate the gga-miR-6577-5p targeted gene Btrc by functioning as a competitive endogenous RNA (ceRNA) for gga-miR-6577-5p, thereby promoting the expression of *Btrc* and facilitating lncRNA-directed PGC development.

## 2. Materials and Methods

### 2.1. Ethics Approval

Animal experiments were approved by the Institutional Animal Care and Use Committee of the Yangzhou University Animal Experiments Ethics Committee (permit number: SYXK [Su] IACUC 2012-0029). All experimental procedures were performed in accordance with the Regulations for the Administration of Affairs Concerning Experimental Animals approved by the State Council of the People’s Republic of China.

### 2.2. Antibody and Reagent

Commercially available antibodies were used: anti-DDX4/*Cvh* (ab13840, Abcam, Cambridge, UK); Goat Anti-Mouse IgG (Cy3 labeled, Bio-Synthesis, Inc., Louisville, KY, USA; dilution ratio:1:100); anti-NANOG and anti-OCT4 (Abcam, dilution ratio 1:100); anti-SSEA (BioLegend, San Diego, CA, USA, dilution ratio 1:100); anti-integrin α6 and anti-integrin β1 (BioLegend, dilution ratio 1:100).

Dulbecco’s modified Eagle medium (DMEM, 41965062) and fetal bovine serum (FBS, 10100-147) from Gibco (Grand Island, NY, USA); FuGENE^®^ HD (E2311) and Dual-Luciferase^®^ Reporter Assay System from Promega (Madison, WI, USA); Leukemia Inhibit Factor from mouse (mLIF, L5158), Fibroblast Growth Factor-Basic human (bFGF, F0291), Stem Cell Human (SCF, S7901); TSA from Sigma (St. Louis, MO, USA); PARIS™ from Ambion (Austin, TX, USA); retinoic acid (R2625) from Sigma.

### 2.3. Experimental Animals

A total of 180 fertilized Rugao yellow eggs were purchased from the Poultry Research Institute of the Chinese Academy of Agricultural Sciences (Yangzhou, China). All eggs were incubated at 37 °C and 75% relative humidity. Tissues of heart, liver, lung, glandular stomach, gizzard, testis, and ovary obtained from 3 adult chickens were harvested for the organ-specific expression analysis.

### 2.4. Cell Culture and Plasmids

In vitro isolation of ESCs required 60 freshly fertilized eggs. ESCs were isolated using blastoderm cells in the embryonic region of stage X fertilized eggs. The isolated blastoderm cells were cultured in DMEM containing 10% FBS, 2% Chicken serum, 10 ng/mL bFGF, 1000 IU/mL LIF, and 5 ng/mL SCF at 37 °C, and 5% CO_2_ saturated humidity for 24 h and then subjected to differential purification of ESC.

Sixty freshly fertilized eggs incubated for 4.5 days were harvested to obtain genital ridges for isolation of PGC. Chicken embryo genital ridges were digested with 0.25% trypsin and 0.05% EDTA for 8 min. The PGCs were purified by repeated differential adherence techniques. The first purification selection was performed after cell culturing for 40 min, and non-adherent cells were transferred to a new culture dish. The supernatant was further cultured at 37 °C and 5% CO_2_ saturated humidity.

Sixty freshly fertilized eggs incubated to 18.5 days were harvested to obtain testes for the isolation of SSC. The testis tissue was digested with collagenase for 30 min, and then with 0.25% trypsin and 0.05% EDTA for 8 min. The SSCs were purified by the repeated differential adherence technique. The first purification selection was performed after cell culturing for 40 min, and non-adherent cells were transferred to a new culture dish. The supernatant was further cultured at 37 °C and 5% CO_2_ saturated humidity.

We prepared plasmid pcDNA3.0-*LncPGCR*. RNAi sequence: *LncPGCR*: sh-1 targets GGATCTGGTTTAATCCCATCG; sh-2 targets GCCATTTCTCATCAGAGTAAG; sh-3 targets GGATCTGGTTTAATCCCATCG. The sequence of miRNA-mimic amplification primers is shown in Table 1.

### 2.5. FACS and RNA-seq

ESCs that had been induced for 4 days were labeled by CVH. The Fuzzy C-Means algorithm (FCM) was used to detect the number of PGCs. Finally, data were analyzed on a Flow cytometer (Becton Dickinson, San Jose, CA, USA) with the FlowJo program.

Using FACSAria SORP to sort ESCs, PGCs and SSCs were labeled with antibodies to ensure that high-purity cells were obtained. Among them, Nanog and Oct4 stem cell surface-specific antigens and molecular markers were used to label ESC, tyrosine kinase receptor C-kit and SSEA-1 to label PGC, and spermatogonial stem cell surface markers integrin α6 and integrinβ1 to label SSC. Total RNA was extracted from ESCs, PGCs, and SSCs, using the TRNzol reagent (Invitrogen, Carlsbad, CA, USA) according to the manufacturer’s instructions. RNA degradation and contamination were monitored on 1% agarose gels. RNA purity was checked using the NanoPhotometer^®^ spectrophotometer (IMPLEN, CA, USA). RNA concentration was measured using Qubit^®^ RNA Assay Kit in Qubit^®^ 2.0 Fluorometer (Life Technologies, Carlsbad, CA, USA). RNA integrity was assessed using the RNA Nano 6000 Assay Kit of the Bioanalyzer 2100 system (Agilent Technologies, Santa Clara, CA, USA). Sequencing library construction was conducted by using the TruSeq PE Cluster Kit v3-cBot-HS (Illumina, San Diego, CA, USA) according to the manufacturer’s instructions. The libraries were sequenced on an Illumina Hiseq 2000 platform. All sequence data are available in SRA with the Accession No. SRR12145635, SRR12145634, and SRR12145633.

### 2.6. Isolation of Nuclear and Cytoplasmic RNA

Operations were performed according to the manufacturer’s protocols of PARIS™ system Protein and RNA Isolation System: “add 500 μL Cell Disruption Buffer to every 10^7^ PGCs cells, and incubate on ice for 10 min; After centrifugation at 500× *g* for 5 min at 4 °C, the supernatant is cytoplasm and the precipitate is the nucleus.” To assess any cross-contamination between cytoplasmic and nuclear fractions, the levels of GAPDH (a specific cytoplasmic protein marker) and U1 (a nuclear protein marker) were examined in the two fractions.

### 2.7. Chicken Embryo Vascular Injection

Eggs were swabbed with alcohol swabs before injection using tweezers to open a round hole with a diameter of 1–1.5 cm. After finding the position of the embryo under the stereomicroscope, ophthalmic tweezers were used to gently lift the outer membrane to expose the embryonic blood vessel. Using a micropipette, the lentiviral vector was filled with a final polybrene concentration of 8 ng/µL or the pcDNA 3.0-PGCR vector wrapped with PEI into glass injection needles and injected into the blood vessels of the chicken embryos. After the injection, 20 μL of penicillin was added to the injection site and then cross-sealed with medical tape to continue incubation. 

### 2.8. qRT-PCR

Total RNA was extracted using Trizol (TIANGEN, Beijing, China) and reverse-transcribed into cDNA with the Quantscript RT Kit (TIANGEN). Gene expression was determined using an ABI PRISM 7500 fluorescent quantitative PCR instrument (Applied Biosystems, Carlsbad, CA, USA). qRT-PCR primers are shown in Table 2, with the internal reference gene: β-actin.

### 2.9. Indirect Immunoinfluscent Assay (IFA)

ESCs that had been induced for 4 days were fixed with 4% paraformaldehyde for 30 min and then treated with 0.5% TritonX-100 for 15 min. After blocking with 10% fetal bovine serum at 37 °C for 2 h, the cells were incubated in anti-*CVH* (1:500, Abcam) for 12 h at 4 °C; After multiple washes by PBST, cells were incubated with corresponding secondary antibodies (1:400, Abcam) for 2 h at room temperature. After DAPI (5 ng/uL) staining, slides were plated with glycerin, and the image was sealed. The FV1000 laser scanning confocal microscope (Olympus, Tokyo, Japan) was used to observe the samples.

### 2.10. Dual-Luciferase Assays

The amplified promoter of *LncPGCR* and different deletion fragments of the *LncPGCR* promoter were subcloned into the firefly plasmids in the pGL3Basic luciferase vector (Promega). Different length PCR amplification primers of the *LncPGCR* promoter region are shown in Supplementary Table S1. TCF7L2 binding site mutation primers are shown in Supplementary Table S2. Finally, a Dual-Luciferase Reporter Assay System (Promega) was used to evaluate luciferase activity. Each procedure of these experiments was repeated independently three times. 

### 2.11. Data Analysis

Relative gene expression was calculated using the 2^−ΔΔCt^ method after PCR [28]. All experiments were performed in triplicate, and the data are expressed as mean ± standard error. Significant differences between the groups were determined with two-sample *t*-tests in SPSS 18.0. (*, *p* < 0.05, significant difference. **, *p* < 0.01, extremely significant difference). GraphPad Prism7 software (GraphPad Software, San Diego, CA, USA) was used for mapping.

## 3. Results

### 3.1. Searching for LncRNAs Involved in the Development of PGCs

**Figure 2:**
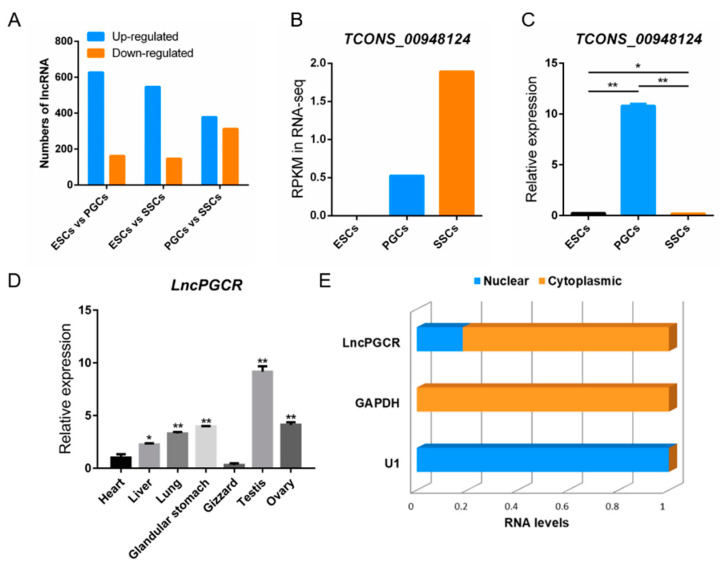
*LncPGCR*, a PGC specific LncRNA in Chicken, is mainly expressed in the cytoplasm. (**A**) LncRNAs differentially expressed in ESCs vs PGCs, ESCs vs SSCs, and PGCs vs SSCs as examined by RNA-seq. (**B**) RNA-seq measurement showing the transcript expression levels of *LncPGCR* in ESCs, PGCs, and SSCs. (**C**) Validation of mRNA expression levels of *LncPGCR* by qRT-PCR. Values are means ± SEM. *n* = 3. (**D**) Relative expression levels of *LncPGCR* in adult chicken tissues as measured by qRT-PCR after normalization to β-actin. * *p* < 0.05, and ** *p* < 0.01. Values are means ± SEM. *n* = 3. (**E**) Relative expression levels of *LncPGCR* in the nucleus and cytoplasm of PGCs as measured by qRT-PCR after normalization to β-actin. * *p* < 0.05, and ** *p* < 0.01. Values are means ± SEM. *n* = 3.

To identify lncRNAs involved in the regulation of PGCs development, we performed an in-depth analysis of differentially expressed lncRNAs by RNA-seq (Figure 2A,B). A comparison of the lncRNA expression profiles in chicken ESCs, PGCs, and SSCs showed that 15 lncRNAs were differentially expressed in PGCs (Table 3, Table S3). We focused on LncRNA (*LncPGCR*, TCONS_00948124), since it was strongly related to germ cell differentiation and because, based on computational prediction algorithms, there were potential interactions of *LncPGCR* with several miRNAs including gga-miR-6577-5p. Then, we evaluated the level of *LncPGCR* expression in ESCs, PGCs, and SSCs by qRT-PCR, and results indicated that *LncPGCR* was also up-regulated in PGCs (*p* < 0.01) (Figure 2C). Chicken tissue expression profiling revealed that *LncPGCR* was highly expressed in gonads (*p* < 0.01) (Figure 2D), suggesting that *LncPGCR* was likely to be involved in PGC development.

We found that *LncPGCR* was expressed in both the nucleus and the cytoplasm of the PGCs, but the expression level in the cytoplasm was much higher than that in the nucleus, which indicated that the mechanism of *LncPGCR* action mainly existed in the post-transcriptional level (Figure 2E). These data demonstrated that *LncPGCR* is highly expressed in the cytoplasm of PGCs and might be critical to the development of PGCs in chickens.

### 3.2. LncPGCR Promotes Development of PGCs In Vitro and In Vivo

**Figure 3:**
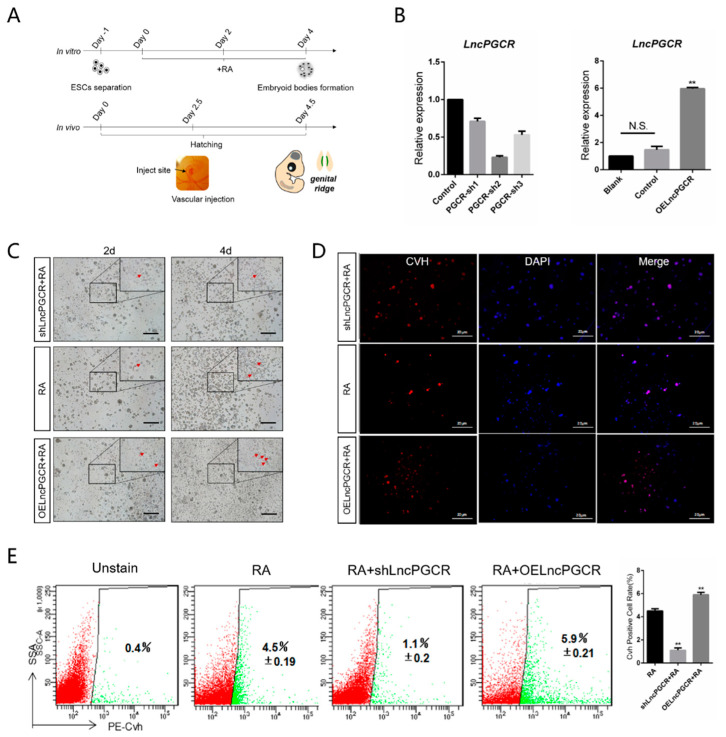
Ectopically expressed *LncPGCR* promotes PGCs formation in vitro and in vivo. (**A**) Schematic diagram of *LncPGCR* function verification in vivo and in vitro. (**B**) Activity verification of *LncPGCR* overexpression and knockdown expression vectors. (**C**) Morphological changes of cells after overexpression or knockdown of *LncPGCR* in the RA induction model. (**D**) Indirect immunoinfluscent analysis of *Cvh* (red) on day 4 after overexpression or knockdown with *LncPGCR* in the RA induction model in vitro. Scale bar: 20 μm. (**E**) The proportion of d4 PGC-like cells (*Cvh*+) was detected by flow cytometry analysis.

To define the function of *LncPGCR* on PGC development, we transfected the *LncPGCR* overexpression/knockdown vectors based on the retinoic acid (RA) induction model and observed the morphology of ESC cells (Figure 3A,B). After the over-expression of *LncPGCR*, we observed a large number of embryonic bodies and PGC-like structures in ESCs after 2–4 days of RA induction. However, knockdown *LncPGCR* triggers a significant decline in PGC-like development (Figure 3C).

Indirect immunoinfluscent assay and flow cytometry analysis confirmed that *LncPGCR* promotes the development of PGCs. Specifically, the percentage of *Cvh*^+^ PGCs at 4d induction in the RA model was significantly higher in the *LncPGCR* overexpression group than in the control group or the *LncPGCR* interference group (Figure 3D,E). qRT-PCR results indicated that overexpression of *LncPGCR* accelerates the down-regulation of the totipotent gene *Nanog* to promote cell differentiation (*p* < 0.05) (Supplementary Figure S1A). At the same time, overexpression of *LncPGCR* promotes the expression of the PGC marker genes *Cvh* and *C-kit* (*p* < 0.01). In contrast, *LncPGCR* knockdown led to significant increases in *Nanog* expression, and the expression of *Cvh* and *C-kit* showed significant downregulation.

To further verify the function of *LncPGCR* in the development of PGC, we injected the PGCR-sh2 lentiviral vector and pcDNA3.0-PGCR vector into the E2.5d chicken embryonic blood vessels. qRT-PCR results showed that after knocking down *LncPGCR*, the expression of *Nanog* was increased (*p* < 0.01), while the expression of *Cvh* and *C-kit* were decreased (*p* < 0.01) (Supplementary Figure S1B). Flow cytometry analysis confirmed that the development of PGCs was suppressed after the knockdown of *LncPGCR* (1% ± 0.19) (Supplementary Figure S1C), which was consistent with the results of induction in vitro. Hence, we confirmed that *LncPGCR* promotes the development of PGCs.

### 3.3. Deacetylation Downregulate LncPGCR Expression

**Figure 4:**
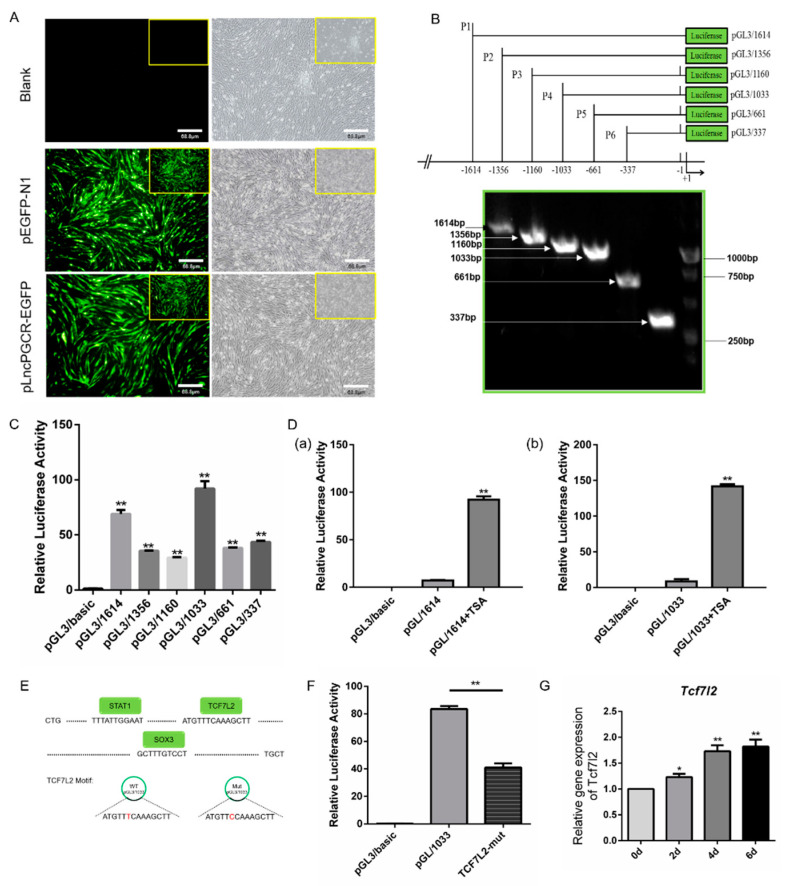
Histone acetylation, and TCF7L2 regulate the expression of *LncPGCR*. (**A**) Qualitative activity detection of *LncPGCR* promoter long fragment (100×). Scale bar: 68.8 μm. (**B**) Schematic diagram of vector construction for *LncPGCR* promoter. (**C**) Dual luciferase analysis of the core region of *LncPGCR*’ promoter. * *p* < 0.05, and ** *p* < 0.01 compared with the control pGL3/BASIC vector. Values are means ± SEM. *n* = 3. (**D**) Levels of *LncPGCR* promoter activity in DF1 cells after adding TSA co-transfected with pGL3/1614(a) or pGL3/1033(b). (**E**) Top: the location of STAT1, TCF7L2, and SOX3 in the core region of the *LncPGCR* promoter; Bottom:diagram of TCF7L2 transcription factor binding site mutation. (**F**) Levels of *LncPGCR* promoter activity in DF1 cells after mutation of the TCF7L2 binding site. * *p* < 0.05, and ** *p* < 0.01 compared with the control pGL3/BASIC vector. Values are means ± SEM. *n* = 3. (**G**) qRT-PCR of Tcf7l2 expression during the induction of ESCs into PGCs in vitro. Values are means ± SEM. *n* = 3.

To elucidate the mechanisms regulating *LncPGCR* expression in the PGC development, we cloned the promoter of *LncPGCR* and replaced the CMV promoter of pEGFP-N1 (Figure 4A). We ligated a series of promoter fragments into the pGL3-basic vector and transfected into DF1 cells to identify the core region of the *LncPGCR* promoter with a dual luciferase reporter system (Figure 4B). The dual-luciferase assay revealed that the −1033~−661 bp region was the core active region of the *LncPGCR* promoter (Figure 4C).

Epigenetic determinants are known to establish and maintain specific patterns of transcription through modulation of DNA methylation and posttranslational modifications of core histones during development [12,29,30,31]. Histone deacetylase (HDAC) is a class of enzymes that removes acetyl groups from a histone, allowing the histones to wrap the DNA more tightly [29]. TSA can bind to HDAC, inhibit histone deacetylation, and promote gene expression. To assess whether epigenetic factors affected *LncPGCR* expression, we add TSA (1 μmol/L) to DF-1 cells transfected with pGL/1614. The dual-luciferase assay verified that TSA significantly increase the *LncPGCR* promoter activity (*p* < 0.01) (Figure 4D). Together, our results indicated that deacetylation are involved in the downregulation of *LncPGCR*.

### 3.4. Transcription Factor TCF7L2 Regulates the Expression of LncPGCR

Transcription factors (TFs) play a pivotal role in the regulation of gene expression [30,31]. To further explore the regulators of the *LncPGCR* promoter, we predicted the transcription factors which might bind with the promoters of *LncPGCR* by the JASPAR databases. There are binding sites for transcription factors STAT1, TCF7L2, and Sox3 in the core region of the *LncPGCR* promoter, and TCF7L2 had the highest score (Figure 4E). Moreover, the dual-luciferase assay revealed that mutation of the TCF7L2 binding site significantly reduced the *LncPGCR* promoter activity (*p* < 0.01), indicating that TCF7L2 is a positive regulator of the *LncPGCR* promoter (Figure 4F). Interestingly, the transcription factor TCF7L2 was up-regulated during the induction of ESCs into PGCs *in vitro*, consistent with the expression trend of *LncPGCR* (Figure 4G). All these results indicated that TCF7L2 served as a positive regulator of *LncPGCR*.

### 3.5. LncPGCR Functions as a ceRNA for gga-miR-6577-5p

**Figure 5:**
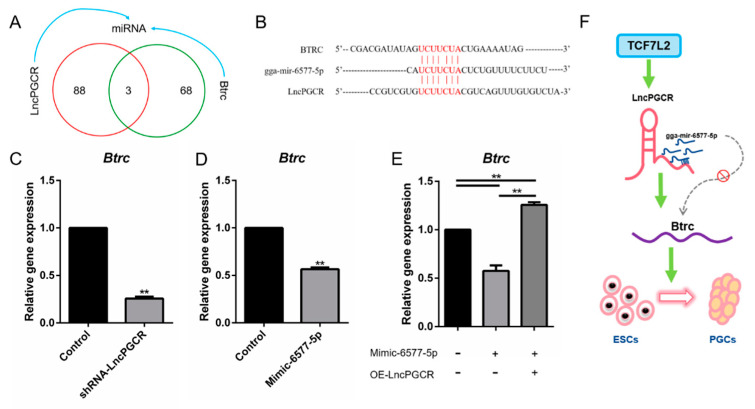
*LncPGCR* and gga-miR-6577-5p regulate PGC development through their target gene *Btrc*. (**A**) Screen potential miRNA targets of *LncPGCR* and *Btrc* by analyzing crosstalk results of RNA-seq and miRDB. (**B**) Sequence alignment of gga-miR-6577-5p with the putative binding sites of *LncPGCR* and *Btrc*. (**C**) *LncPGCR* can up-regulate *Btrc* expression as measured by qRT-PCR. * *p* < 0.05, and ** *p* < 0.01 compared with control. Values are means ± SEM. *n* = 3. (**D**) gga-miR-6577-5p can inhibit *Btrc* expression as measured by qRT-PCR. * *p* < 0.05, and ** *p* < 0.01 compared with control. Values are means ± SEM. *n* = 3. (**E**) Up-regulated gga-miR-6577-5p in DF1 cells, which stably over-expressed *LncPGCR*, largely reversed the favorable effects of *LncPGCR* on *Btrc* expression. * *p* < 0.05, and ** *p* < 0.01 compared with control. Values are means ± SEM. *n* = 3. (**F**) Model proposed to explain the influence of *LncPGCR* upon the PGCs development.

Salmena [32] proposed a competitive endogenous RNA (ceRNA) hypothesis, that mRNA, transcribed pseudogenes, and lncRNA act as natural miRNA sponges by sharing one or more miRNA response elements in a large-scale regulatory network to inhibit miRNA function, thereby affecting the occurrence and development of diseases. To test if *LncPGCR* stimulates development of PGCs by modulating the miRNA function, we evaluated the miRNA binding sites on *LncPGCR* using DIANA TOOLS and found 91 miRNA binding sites. Cis-tether prediction found that the target gene of *LncPGCR* is *Btrc* (Table 3). Therefore, we used miRDB to predict the miRNAs that have binding sites for *Btrc* in chicken and found 71 miRNAs. Three miRNAs (gga-mir-6577-5p, gga-mir-6625-5p, and gga-mir-3539) with binding sites to *Btrc* and *LncPGCR* were screened by Venn analysis (Figure 5A). Based on their bioinformatics scores, we chose gga-miR-6577-5p to verify the assumption (Figure 5B). qRT-PCR results showed that *LncPGCR* promoted the expression of *Btrc*, while gga-miR-6577-5p inhibited its expression (Figure 5C,D), confirming that *Btrc* is a common target gene of *LncPGCR* and gga-miR-6577-5p. Strikingly, the co-transfection of Mimic-6577-5p and pcDNA3.0-*LncPGCR* reversed the transcription-inhibitory role of gga-miR-6577-5p (Figure 5E).

## 4. Discussion

In this present study, we identified several LncRNAs that are aberrantly expressed in PGCs. Among them, *LncPGCR* was the most upregulated in PGCs. Here, we showed that *LncPGCR* was highly expressed in PGCs and gonad tissues, and high *LncPGCR* expression was closely associated with the development of PGCs. We also confirmed that the overexpression of *LncPGCR* can promote PGC development, both in vivo and *in vitro*, and that TCF7L2 mediated upregulation of *LncPGCR*. Mechanistically, we showed that *LncPGCR* directly interacted with gga-miR-6577-5p at the recognized sites. *LncPGCR* exerted its function on PGCs in large part due to its role as a ceRNA for gga-miR-6577-5p, and subsequently to initiate the Wnt/*Btrc* signaling pathway. These findings suggest that *LncPGCR* may exert a facilitating function and play a key role in PGC’ development.

Epigenetic modification can regulate the differential expression of *LncPGCR*. Previous studies have shown that histone acetylation can change the structure of nucleosomes, keep the chromatin development open, and promote the binding of transcription factors to chromosomal DNA, which is conducive to gene transcription and expression [33]. What is interesting to us is that the transcription factor TCF7L2 can target the regulation of *LncPGCR* expression. Transcription factor 7-like-2 (TCF7L2), also known as T-cell transcription factor 4 (TCF-4), is a transcription factor containing a DNA-binding domain [34]. Previous studies have shown that TCF7L2 is an important component of the Wnt signaling pathway [35]. Moreover, our previous studies have verified that Wnt signaling pathways are involved in regulating the differentiation of ESCs to SSCs [19]. It was also found that TCF7L2 can affect the development of PGCs as a key downstream transcription factor of the Wnt signaling pathway(data not published), suggesting that *LncPGCR* is regulated by WNT signals to participate in the development of PGCs.

LncRNA can be used as a molecular sponge of endogenous miRNA, and its molecular regulatory mechanism through binding to miRNA is the ceRNA mechanism [36,37]. This mechanism has received attention since it was proposed and it represents a new model of gene expression regulation. Wang found that linc-ROR can regulate human ESC maintenance and differentiation, and the subsequent sequence and function analysis found that because linc-ROR shared miRNA response elements with core transcription factors such as OCT4, SOX2, and NANOG, Linc-ROR acts as a sponge during ESC cell differentiation, preventing these core transcription factors from miRNA-mediated inhibition [38]. Here, our results suggest a novel model whereby *LncPGCR* promotes PGC development by acting as a ceRNA for gga-miR-6577-5p (Figure 5F).

In this study, we identified that *Btrc* is a potential target of gga-miR-6577-5 and *LncPGCR*. *Btrc* (also known as SCF^β-TrCP^) is a key downstream gene of the WNT pathway. As a member of the F-box family, it encodes an F-box protein(β-TrCP), and the coding protein β-TrCP of *Btrc* is one of the basic members of SCF^β-TrCP^ [39]. In our research on *LncPGCR*, we found that its base sequence is complementary to the *Btrc*. Therefore, we speculate that *LncPGCR* may affect the expression of *Btrc* and ultimately affect the development of PGCs. Here, we confirmed that *LncPGCR* can promote the expression of *Btrc*. In addition, our results also demonstrated that gga-miR-6577-5 functioned as a suppressor of PGC development by directly targeting *Btrc*.

## 5. Conclusions

In sum, our results indicate that *LncPGCR* functions as a potential stimulator of PGCs development through the gga-miR-6577-5/*Btrc* axis. Our findings may offer a novel positive regulator and more efficient induction methods for PGCs development.

## Figures and Tables

**Table 1:** pri-miR primer sequences.

Name	Sequence (5′ to 3′)
mir-6577-5p-F	CCGGAATTCGGCTGCTAAGGTATAGCACAGCTGTG
mir-6577-5p-R	CCGGGATCCCCTTACCCAGCAGAGCAACTCAG

**Table 2:** Primer information for qRT-PCR.

Gene	Primer Sequence
β-actin	F: CAGCCATCTTTCTTGGGTAT
	R: CTGTGATCTCCTTCTGCATCC
Gapdh	F: TCAAATGGGCAGATGCAGGT
	R: TCAGCAGCAGCCTTCACTAC
U1	F: ACATGGTGTACAACAAGCGC
	R: CTCACCGCTCATCGTATCGG
LncPGCR	F: TGGATGGTCAAAGGGATGCC
	R: ATGAGAAATGGCTGTGGGGG
Cvh	F: CCACGGCTATTTCACACCTCTG
	R: GCTCTTGGCAAGCATCCGTA
C-kit	F: GCGAACTTCACCTTACCCGATTA
	R: TGTCATTGCCGAGCATATCCA
Nanog	F: TGGTTTCAGAACCAACGAATGAAG
	R: TGCACTGGTCACAGCCTGAAG
Btrc	F: TCAAATGGGCAGATGCAGGT
	R: TCAGCAGCAGCCTTCACTAC
gga-mir-6577-5p	F: acggcgcgCATCTTCTACTCTGTTTTCTTCT
	R: CAGTGCGTGTCGTGGAGT

**Table 3:** Differences in Specific Expression Related to Germ Cell Differentiation in PGCs lncRNA.

LncRNA	Target Gene	KEGG
TCONS_00612668	BCL9L	--
TCONS_00765266	Lef-1	Wnt signaling pathway, Adherens junction, Melanogenesis
TCONS_00874170	BMP4	TGF-beta signaling pathway, Hedgehog signaling pathway
TCONS_00072199	LIG4	Non-homologous end-joining
TCONS_00362261	MAPK1	TGF-beta signaling pathway, MAPK signaling pathway
TCONS_00362344	TBX3	--
TCONS_00612668	DDX6	RNA degradation
TCONS_00659989	NR2E1	--
TCONS_00946310	TRIM8	TGF-beta signaling pathway
TCONS_00580256	SALL4	--
TCONS_00612668	PHLDB1	--
TCONS_00947438	TCF7L2	Wnt signaling pathway, Adherens junction, Melanogenesis
TCONS_00978804	ACVR2A	TGF-beta signaling pathway
TCONS_00948124	BTRC	Wnt signaling pathway, Hedgehog signaling pathway, Oocyte meiosis
TCONS_00627195	IGF2BP1	--

## Data Availability

The data presented in this study are available on request from the corresponding author.

## Supplementary Materials

The following are available online at https://www.mdpi.com/2076-2615/11/2/292/s1, Figure S1: *LncPGCR* regulated the expression of the totipotent gene *Nanog* and PGC marker genes to promote PGCs development (**A**). qRT-PCR of Nanog, Cvh, and C-kit expression after overexpression or knockdown with LncPGCR in vitro. * *p* < 0.05, and ** *p* < 0.01 compared with untreated ESCs. Values are means ± SEM. *n* = 3. (**B**). qRT-PCR of Nanog, Cvh, and C-kit expression after overexpression or knockdown with LncPGCR in vivo. * *p* < 0.05, and ** *p* < 0.01 compared with the untreated genital ridge. Values are means ± SEM. *n* = 3. (**C**). The proportion of PGCs-like cells(Cvh^+^) in the genital ridge on E4.5d detected by flow cytometry analysis. Table S1: *LncPGCR* promoter region different length PCR amplification primers, Table S2: Transcription factor TCF7L2 binding site mutation primer. Table S3: The transcript expression levels of PGCs lncRNA related to germ cell differentiation in ESCs, PGCs, and SSCs.
