# Supplementary material for: Long Noncoding RNA LncPGCR Mediated by TCF7L2 Regulates Primordial Germ Cell Formation in Chickens
Source: Animals (Basel). 2021 Jan 24;11(2):292. doi: 10.3390/ani11020292 (PMC7912682; doi:10.3390/ani11020292)
Supplement: Supplementary file 1 [file animals-11-00292-s001.pdf]

animals

MDPI

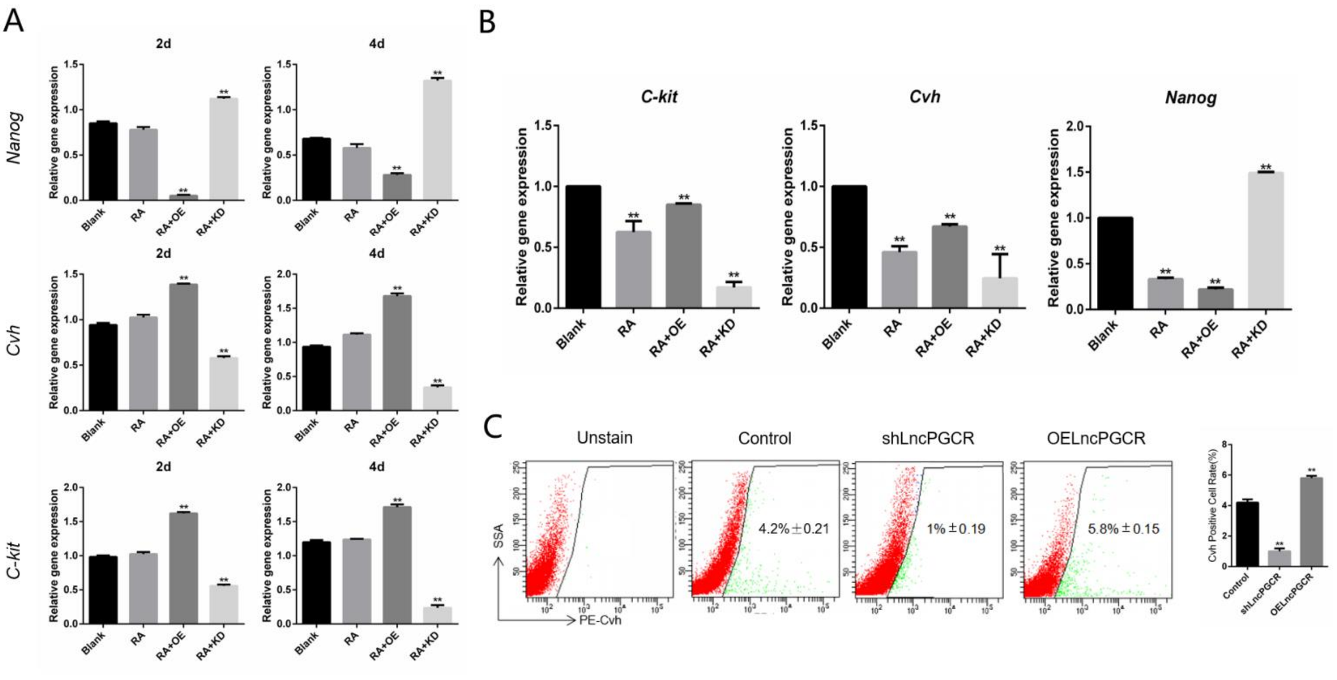

Figure S1. *LncPGCR* regulated the expression of the totipotent gene *Nanog* and PGC marker genes to promote PGCs development **(A)**. qRT-PCR of Nanog, Cvh, and C-kit expression after overexpression or knockdown with LncPGCR in vitro. * $p < 0.05$, and ** p < 0.01 compared with untreated ESCs. Values are means ± SEM. n=3. **(B)**. qRT-PCR of Nanog, Cvh, and C-kit expression after overexpression or knockdown with LncPGCR in vivo. * $p < 0.05$, and ** p < 0.01 compared with the untreated genital ridge. Values are means ± SEM. n=3. **(C)**. The proportion of PGCs-like cells(Cvh^{+}) in the genital ridge on E4.5d detected by flow cytometry analysis.

Table S1. LncPGCR promoter region different length PCR amplification primers.

Name	Primer Sequence	Size	Enzyme Sites	Usage
P1	CCCTACGTACCTTTGGGTTCAGAGTCCTG	1614	XhoI	pLncPGCR-EGFP
P2	CGGGGTACCCCTTGAGGGCTTTCCTTTGC	1356	KpnI	pGL3/1356
P3	CGGGGTACCGGTAGTCCCGATGCCAGTCC	1160	KpnI	pGL3/1160
P4	CGGGGTACCGGTTTCCACTGAACACCCTC	1033	KpnI	pGL3/1033
P5	CGGGGTACCATCCCAGCTTTGTCCTTGCT	661	KpnI	pGL3/661
P6	CGGGGTACCCCCAACCCGAGGAGCGAAGA	337	KpnI	pGL3/337
P0	CCGCTCGAGATCAAAGCGATTTCCCTGTA		SnaBI	pLncPGCR-EGFP

Table S2. Transcription factor TCF7L2 binding site mutation primer.

	Name	Primer Sequence
TCF7L2	F-Kpn I	TGATCTTAATCAACCATGTT**G**CAAAGCTTCATACCGTAAGCACA
	TCF7L2-R1	TGTGCTTACGGTATGAAGCTTTG**C**AACATGGTTGATTAAGATCA
	TCF7L2-F1	TGATCTTAATCAACCATGTT**G**CAAAGCTTCATACCGTAAGCACA
	R-Xho I	GCTTACTTAGATCGCAGATCTCGAGATCAAAGCGATTTCCCTGTA

Table S3. The transcript expression levels of PGCs lncRNA related to germ cell differentiation in ESCs, PGCs, and SSCs.

lncRNA	ESCs_FPKM	PGCs_FPKM	SSCs_FPKM	*P* values
TCONS_00612668	0.304065	5.2481	2.91182	0.0268936
TCONS_00765266	35.8417	0.0421958	0	2.90E-06
TCONS_00874170	0	0.564523	0.0374735	0.00901045
TCONS_00072199	7.20163	0.0787197	0	0.000343457
TCONS_00362261	37.9311	1.1473	6.82561	0.0297688
TCONS_00362344	22.9197	0.187924	0	0.000404435
TCONS_00612668	0.304065	5.2481	2.91182	0.0268936
TCONS_00659989	22.9598	0.270672	1.37532	0.00405561
TCONS_00946310	1.28279	0.0733322	0	0.0338241

TCONS_00580256	28.7449	0.379114	0	0.00588496
TCONS_00612668	0.304065	5.2481	2.91182	0.0268936
TCONS_00947438	5.34547	0.610657	0.114237	3.90E-06
TCONS_00978804	0	0.105411	2.85304	0.0148691
TCONS_00948124	0	0.524497	1.89062	0.00672651
TCONS_00627195	32.6283	0.0533638	0	1.72E-08
